# Exogenous ketone supplementation: an emerging tool for physiologists with potential as a metabolic therapy

**DOI:** 10.1113/EP090430

**Published:** 2022-12-19

**Authors:** Kaja Falkenhain, Hashim Islam, Jonathan P. Little

**Affiliations:** ^1^ School of Health and Exercise Sciences University of British Columbia Okanagan Kelowna British Columbia Canada

**Keywords:** cardiovascular disease, cognitive function, inflammation, integrative physiology, ketone monoester, ketosis, metabolic health, metabolism, nutrition, type 2 diabetes

## Abstract

Exogenous oral ketone supplements, primarily in form of ketone salts or esters, have emerged as a useful research tool for manipulating metabolism with potential therapeutic application targeting various aspects of several common chronic diseases. Recent literature has investigated the effects of exogenously induced ketosis on metabolic health, cardiovascular function, cognitive processing, and modulation of inflammatory pathways and immune function. This narrative review provides an overview of the integrative physiological effects of exogenous ketone supplementation and highlights current challenges and future research directions. Much of the existing research on therapeutic applications – particularly mechanistic studies – has involved pre‐clinical rodent and/or cellular models, requiring further validation in human clinical studies. Existing human studies report that exogenous ketones can lower blood glucose and improve some aspects of cognitive function, highlighting the potential therapeutic application of exogenous ketones for type 2 diabetes and neurological diseases. There is also support for the ability of exogenous ketosis to improve cardiac metabolism in rodent models of heart failure with supporting human studies emerging; long‐terms effects of exogenous ketone supplementation on the human cardiovascular system and lipid profiles are needed. An important avenue for future work is provided by research accelerating technologies that enable continuous ketone monitoring and/or the development of more palatable ketone mixtures that optimize plasma ketone kinetics to enable sustained ketosis. Lastly, research exploring the physiological interactions between exogenous ketones and varying metabolic states (e.g., exercise, fasting, metabolic disease) should yield important insights that can be used to maximize the health benefits of exogenous ketosis.

## Abstract (highlights)

- **What is the topic of this review?**
  The integrative physiological response to exogenous ketone supplementation.
- **What advances does it highlight?**
  The physiological effects and therapeutic potential of exogenous ketones on metabolic health, cardiovascular function, cognitive processing, and modulation of inflammatory pathways and immune function. Also highlighted are current challenges and future directions of the field.

## 1 INTRODUCTION

The potential for ketosis to improve health and/or enhance physical performance has gained popularity among researchers, industry and the general public. Ketosis is characterized by a state of elevated blood ketones, often measured by assessing the concentration of blood β‐hydroxybutyrate (β‐OHB) – the most abundant and stable ketone body in circulation. Ketone bodies are produced endogenously from free fatty acids by the liver during times of limited glucose availability (e.g., starvation, carbohydrate restriction) and serve as an alternative fuel source for the brain and peripheral tissues (Newman & Verdin, 2017).

The recent development and use of exogenous ketone supplements has enabled researchers to investigate the direct effects of elevated ketones while mitigating the confounding influence of the complex metabolic processes that occur simultaneously with elevated ketone concentrations during endogenous ketosis (Poff et al., 2020). Aside from further exploring the metabolic effects of exogenously induced ketosis, emerging research now supports the role of ketones as hormone‐like signalling molecules (Newman & Verdin, 2017; Puchalska & Crawford, 2021). This raises the intriguing possibility that elevating blood ketones exogenously or endogenously could have widespread systemic effects impacting not only metabolic tissues but also immune cells, smooth muscle cells and neurons. The purpose of this narrative review is to provide an update on the integrative physiological responses to exogenous ketone supplementation in humans and to highlight the potential of exogenous ketone supplements as a metabolic therapy.

## 2 BACKGROUND AND HISTORICAL CONTEXT

Initial interest in the state of ketosis dates back to the early 20th century, when the ketogenic diet originated as a treatment for epilepsy. Although fasting was known to be an effective treatment for epilepsy for centuries, it was noticed that the rise in ketone bodies observed during fasting or starvation was also apparent in response to a very low carbohydrate diet (Woodyatt, 1921). It was proposed that the benefits of fasting could be obtained through use of a ketogenic diet consisting of a very low intake of carbohydrate and a high proportion of fat, which could mimic aspects of starvation by shifting whole‐body metabolism towards fat oxidation and ketone body production (Wilder, 1921). Following this discovery, the ketogenic diet became a widely used and documented treatment for epilepsy until the discovery of efficacious pharmacological drugs in the 1930s. Around the same time, the use of a calorie‐restricted, high fat, very low carbohydrate diet in patients with diabetes was pioneered (Newburgh, 1920). However, these findings were largely forgotten with the discovery and subsequent widespread availability of insulin in the 1930s.

Research on the metabolic effects of ketosis continued throughout subsequent decades, often with study designs that infused ketones and measured the subsequent impacts on metabolism (Robinson & Williamson, 1980). In fact, the 1970s brought about the development of the first intravenously infused ketone esters that were trialled for their use as parenteral nutrition (Brunengraber, 1997). These included esters of 1,3‐butanediol and β‐OHB as the immediate predecessor to today's ketone monoesters.

The development of the first commercially available exogenous ketone supplements that could be consumed orally (Clarke et al., 2012) obviated the need for invasive procedures (i.e., infusion of exogenous ketones) and prolonged dietary modification (e.g., fasting, ketogenic diet) to achieve ketosis. A number of exogenous ketone supplements are now available on the market that broadly fall into two main categories: ketone salts and ketone esters. Ketone esters can be further divided into mono‐ and diesters or based on whether they contain β‐OHB and/or precursors thereof. Ketone salts consist of β‐OHB bound to a mineral (e.g., sodium, potassium, magnesium), whereas ketone esters are ketones bonded with a precursor molecule such as 1,3‐butanediol (Stubbs et al., 2017). Generally, ketone esters are more potent at raising blood ketones than ketone salts and lead to fewer adverse side effects (e.g., less gastrointestinal discomfort) (Falkenhain et al., 2022).

The following sections will highlight the impact of exogenous ketone supplementation in humans, with a focus on physiological effects with therapeutic potential in a variety of disease states.

## 3 GLUCOSE‐LOWERING EFFECT OF EXOGENOUS KETONES AND ASSOCIATED MECHANISMS

Although the glucose‐lowering properties of β‐OHB were observed in infusion studies early‐on (Neptune, 1956), initial research employing modern‐day exogenous ketone supplements mostly focused on testing the potential of ketosis to enhance exercise performance (Cox et al., 2016; O'Malley et al., 2017). Informed by the serendipitous observation that blood glucose was lowered during exercise after ingestion of exogenous ketones compared to a non‐carbohydrate‐containing comparator in these studies, evidence supporting the glucose‐lowering properties of exogenous ketones began to accumulate.

Subsequent studies have demonstrated the ability of acute ketone monoester ingestion to lower the rise in plasma glucose following an oral glucose tolerance test in healthy individuals (Myette‐Cote et al., 2018), individuals with obesity (Myette‐Cote et al., 2019), and individuals with impaired glucose tolerance (Nakagata et al., 2021). All of these studies provided the ketone monoester at a dose of 482 mg/kg body mass that elicited average plasma β‐OHB concentrations of ∼2–3 mM. Ketone monoester ingestion provided at 395 mg/kg lean body mass leading to a peak β‐OHB concentration of ∼3.5 mM was also able to lower glucose in a fasted state compared to a calorie‐free placebo in individuals with prediabetes (Bharmal et al., 2021). Extending these acute laboratory‐based findings, 14 days of thrice daily pre‐meal ketone monoester supplementation (36 g/day leading to plasma β‐OHB concentrations of ∼ 2 mM following ketone ingestion) lowered mean daily glucose and postprandial glucose excursions under free‐living conditions in individuals with overweight/obesity (Walsh, Neudorf, et al., 2021). These studies highlight the therapeutic potential of exogenous ketone supplementation for hyperglycaemia‐related conditions such as insulin resistance and type 2 diabetes (T2D) (Walsh, Myette‐Cote, Neudorf, et al., 2020). This hypothesis is further supported by a recent free‐living single‐arm pilot study reporting improved glycaemic control when individuals with T2D consumed a ketone monoester three times per day for 28 days, albeit at a higher dose of 75 g daily leading to average plasma β‐OHB concentrations of ∼3.5 mM (Soto‐Mota et al., 2021). Overall, these findings show that exogenous ketones are capable of both lowering blood glucose in the fasted state and attenuating postprandial glucose excursions, with a recent meta‐analysis suggesting a strong linear relationship between plasma β‐OHB‐exposure (i.e., measures of peak concentration as well as the area under the curve) and the extent of the glucose‐lowering effect (Falkenhain et al., 2022).

**FIGURE 1:**
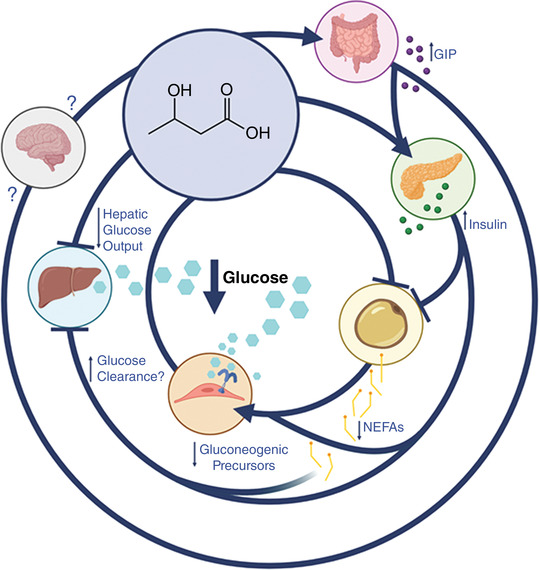
Schematic overview of potential mechanisms underlying the glucose‐lowering effect of exogenous ketones. BDNF, brain‐derived neurotrophic factor; GIP, glucose‐dependent insulinotropic polypeptide; NEFAs, non‐esterified fatty acids; NLRP3; nucleotide oligomerization domain‐like receptor pyrin‐domain containing 3. (Created with BioRender.com)

The mechanisms underlying the glucose‐lowering effect of exogenous ketones have only partially begun to be elucidated (Figure 1). Early on, investigators observed an increase in insulin secretion after infusion of acetoacetate (which also leads to a rise in circulating β‐OHB) that was accompanied by a progressive decrease in blood glucose (Balasse et al., 1970). However, elevated concentrations of insulin were apparent only in the portal vein as peripheral insulin concentration appeared unaltered. Others have similarly found changes in plasma C‐peptide concentrations without a concomitant change in peripheral insulin upon β‐OHB infusion raising total ketone levels to ∼2.5 mM (Miles et al., 1981), whereas a recent meta‐analysis (including 10 studies that measured insulin) reported an acute increase in plasma insulin following ingestion of oral exogenous ketones (Falkenhain et al., 2022). These findings suggest that a direct stimulatory effect on the pancreatic β‐cells leading to insulin secretion is a potential glucose‐lowering mechanism (Madison et al., 1964), which is supported by preclinical work on isolated rat islet cells showing that β‐OHB directly stimulates insulin secretion in the presence (but not absence) of glucose (Biden & Taylor, 1983). However, the glucose‐lowering property of exogenous ketones appears conserved in humans with insulin‐dependent diabetes who lacked insulin secretory capacity (Sherwin et al., 1976) highlighting the existence of alternative glucose‐lowering mechanisms. Furthermore, recent studies have observed an increase in plasma insulin following oral exogenous ketone ingestion but not during intravenous infusion despite both interventions eliciting similar increases in blood β‐OHB concentrations and reductions in blood glucose (Rittig et al., 2020). These findings suggest that the route of administration and/or gut‐derived hormones may play a role in orchestrating the response to exogenous ketones, strongly suggesting the potential of additional insulin‐independent mechanisms mediating the glucoregulatory effects of ketones.

It has repeatedly been demonstrated that exogenous ketones – either intravenously or orally administered – decrease endogenous glucose production (Miles et al., 1981; Svart et al., 2021). Although potentially due, in part, to the above‐discussed stimulation of insulin secretion, other possible explanations include the consistently observed β‐OHB‐induced decrease in non‐esterified fatty acids (NEFAs). β‐OHB can directly inhibit lipolysis via G‐protein‐coupled receptor 109A (GPR109A) present on adipocytes as demonstrated by in vitro studies (Taggart et al., 2005), thereby lowering NEFA availability and subsequently reducing hepatic glucose production via gluconeogenesis (Reaven et al., 2005). Recent findings have also suggested a decrease in gluconeogenic precursors (e.g., l‐alanine) (Soto‐Mota et al., 2022), though others have argued against an overall net decrease in gluconeogenic substrate availability (Miles et al., 1981). Further mechanistic research is needed to confirm the potential contribution of altered gluconeogenic precursor availability to the glucoregulatory effects of ketones. Lower NEFA concentrations could potentially also improve peripheral insulin sensitivity (Santomauro et al., 1999), thereby facilitating enhanced clearance of glucose. In fact, it has been demonstrated that β‐OHB infusion in the context of growth hormone‐induced lipolysis reduced NEFA availability and increased insulin‐stimulated glucose disposal and oxidation in healthy individuals (Høgild et al., 2022). Further research on the effects of ketosis on muscle glucose uptake, oxidation and/or storage is warranted – particularly in the context of different metabolic (e.g., during exercise, fed vs. fasted) or disease (e.g., insulin resistance) states.

Interestingly, exogenous ketone supplements increase plasma glucagon concentrations (Henry et al., 1990; Jensen et al., 2020; Myette‐Cote et al., 2019; Svart et al., 2021). Although little is understood about the contribution of altered plasma glucagon to the glucoregulatory effects of exogenous ketones, it has been suggested that the rise in glucagon counteracts the decrease in glucose. Specifically, if glucagon secretion is suppressed, the suppression of hepatic glucose output will be more pronounced, even in the absence of elevated insulin (Henry et al., 1990). Other consistently observed effects of exogenous ketones include decreased glucagon‐like peptide 1 (GLP‐1) concentrations (Myette‐Cote et al., 2019, 2018; Stubbs et al., 2017) and increased glucose‐dependent insulinotropic polypeptide (GIP) concentrations (Bharmal et al., 2021), both of which may play a role in the effect of orally ingested ketone supplements on glucagon and insulin secretion. However, this effect may be dependent on the metabolic state (i.e., fasted vs. postprandial) given the incretins’ glucose‐dependent mode of action. Furthermore, the physiological significance of reduced GLP‐1 concentration following exogenous ketone ingestion remains unclear, as GLP‐1 is associated with cardiovascular and metabolic benefits and promotes insulin secretion (Baggio & Drucker, 2007). The interpretation of these findings is further complicated by the uncertainty around which form(s) of GLP‐1 is/are reduced (i.e., active vs. reduced inactive forms) based on lack of reporting in primary studies, and the unknown effects of β‐OHB on gastric emptying (Greaves et al., 2021).

Clearly, further research is needed to elucidate the mechanisms contributing to the glucose‐lowering effects of exogenous ketones. This includes the effects of longer‐term/repeated ketone supplementation, as the relative hyperinsulinaemia induced by exogenous ketone supplementation could potentially confound any improvement in glycaemic control by exacerbating insulin resistance, particularly in people predisposed to or diagnosed with metabolic disease. Unravelling the underlying physiology will aid in our understanding and application of exogenous ketones as a potential therapeutic option for hyperglycaemia‐related conditions.

## 4 MODULATION OF INFLAMMATORY PATHWAYS AND IMMUNE FUNCTION BY EXOGENOUS KETONES

Cellular and rodent studies have demonstrated that ketones (in particular, β‐OHB) are able to modulate inflammation by inhibiting the nucleotide oligomerization domain‐like receptor pyrin‐domain containing 3 (NLRP3) inflammasome activation in bone marrow derived macrophages (Youm et al., 2015), leading to decreased downstream production of pro‐inflammatory cytokines interleukin (IL)‐1β and IL‐18. Extending these findings, other rodent and in vitro studies have shown dampened NLRP3 inflammasome‐mediated cytokine production in a number of different disease models. This was demonstrated following β‐OHB treatment of neutrophils and macrophages and via a ketogenic diet in a mouse model of gout (Goldberg et al., 2017), following oral ketone ester administration in lipopolysaccharide (LPS)‐induced sepsis in mice (Soni et al., 2022, Ji et al., 2022), following intraperitoneal β‐OHB injections in mice and β‐OHB treatment of human placental tissue cultures and an animal model of LPS‐induced pregnancy pathology (Hirata et al., 2021), and via 2022 treatment of BV2 cells with β‐OHB in the context of spinal cord injury (Kong et al., 2021).

Furthermore, a ketogenic diet reduced adipose tissue inflammation (Goldberg et al., 2020) and improved T‐cell function (Goldberg et al., 2019; Zhang et al., 2020) in mice, which highlights the need to explore whether exogenous ketones might be able to ameliorate inflammation in obesity. Additionally, it has been suggested that β‐OHB (administered via intraperitoneal or subcutaneous injection in mice) may exert anti‐inflammatory effects directly via GPR109A (Graff et al., 2016), as demonstrated in neuroinflammation associated with Parkinson's disease (Fu et al., 2015) or in alcohol‐induced liver injury (Chen et al., 2018).

The interpretation of these in vitro and rodent studies is complicated by recent translational work showing increased markers of NLRP3 activation following acute ingestion of exogenous ketones in healthy individuals (Neudorf et al., 2019). Similarly, peak IL‐1β concentrations were higher during infusion of β‐OHB compared to placebo or lipids in the context of LPS infusion in healthy individuals (Thomsen et al., 2018). These conflicting results could suggest that potential anti‐inflammatory effects of ketones may be dependent on the presence of basal inflammation. In support of this supposition, 14 days of thrice daily supplementation with exogenous ketones resulted in attenuated LPS‐stimulated monocyte caspase‐1 activation and IL‐1β secretion in individuals with obesity without any changes in fasting cytokine concentrations (Walsh, Neudorf, et al., 2021). These findings may also suggest that changes in cellular anti‐inflammatory actions of exogenous ketones may not always be captured by measuring circulating cytokines, highlighting the importance of coupling measurements of blood cytokine concentrations with the assessment of inflammatory processes occurring at the cellular level in future work.

Intriguingly, a recent study in mice provides evidence that elevated β‐OHB, induced by either a ketogenic diet or exogenous ketone monoester ingestion, provides an alternative fuel for CD4^+^ T‐cells that supports mitochondrial metabolism, increases interferon‐γ production and improves viral clearance survival in response to experimental SARS‐CoV‐2 infection (Karagiannis et al., 2022). These findings, together with studies showing that ketosis may enhance T‐cell function in rodent models (Goldberg et al., 2019; Zhang et al., 2020), suggest that raising blood β‐OHB concentration through consumption of exogenous ketone supplements could modify immune cell bioenergetics to improve acute immune function (e.g., targeted and purposeful activation of immune cells to eliminate pathogens).

Although these results are promising, there is a clear need for more work on the integrative effects of β‐OHB and exogenous ketones on inflammatory pathways and immune function in order to translate findings from animal and cell studies to humans, and to distinguish the effects of β‐OHB on acute immune defence from those on chronic inflammation (i.e., ongoing and persistent low‐level production of pro‐inflammatory cytokines by activated immune cells). The integrated physiological response to exogenous oral ketone consumption (e.g., reduced glucose, increased insulin, reduced NEFAs) likely interacts with any direct (signalling) effects of elevated β‐OHB on immune cells, making interpretation of in vivo human studies challenging.

## 5 EXOGENOUS KETONES AND COGNITIVE FUNCTION

Ketone bodies – particularly acetoacetate and β‐OHB – can cross the blood–brain barrier and serve as an important energy substrate for the brain. Using intravenously infused labelled β‐OHB tracers, it is well‐established that the influx of ketone bodies into the brain increases proportionally to the concentration of ketone bodies present in the circulation (Blomqvist et al., 1995; Hasselbalch et al., 1995). Supported by rodent studies showing improved cognitive performance after ketone monoester feeding (at 30% of daily caloric intake) (Murray et al., 2016) and building on the idea that reliance on ketones for energy may benefit cognitive processing (e.g., during times of excess energy demand or due to metabolic impairments leading to energy insufficiency), initial evidence on the effects of exogenously induced ketosis on cognitive performance in humans has begun to emerge. A case study of a patient living with Alzheimer's disease (AD) found that supplementing with a ketone monoester thrice daily (for a total daily dose of ∼65 g) improved mood, affect, cognitive performance and ability to perform activities of daily living (Newport et al., 2015). These findings were supported by two randomized controlled trials that provided 30 g daily of medium‐chain triglycerides (MCTs) to individuals with mild cognitive impairment, resulting in mildly elevated plasma ketones (∼0.5 mM) and increased brain ketone uptake (Fortier et al., 2021; Roy et al., 2021). The intervention improved some domains of cognitive processing and processing speed (Fortier et al., 2021), which correlated with ketone uptake across the whole brain and in individual fascicles (Roy et al., 2021). Similarly, a recent trial showed a reduction on the Alzheimer's Disease Assessment Scale‐Cognitive Subscale after 30 days of supplementation with 17.3 g of MCTs in patients with mild to moderate AD (Xu et al., 2020). Although these findings are promising, the MCTs used in these trials only mildly elevated circulating ketones and therefore may not have maximized the therapeutic potential of ketosis for cognitive health. In contrast, 30 ml of a ketone monoester (equating to 12 g of β‐OHB) elevated blood β‐OHB to ∼1.8 mM in individuals with obesity, and thrice daily supplementation over 14 days improved aspects of cognition compared to a calorie‐free placebo (Walsh, Caldwell, et al., 2021). Similarly, a recent trial found improvements in working memory performance after infusing β‐OHB (compared to saline as placebo) intravenously in individuals with T2D under conditions that elevated blood β‐OHB to ∼2.4 mM while clamping blood glucose at 7.5 mM (Jensen et al., 2020).

Mechanistically, it is unclear how exogenous ketones improve cognitive processing, particularly aspects related to their role as an alternative fuel source supporting brain energy metabolism. Relatedly, it is also unclear how different concentrations of blood ketones (e.g., physiological vs. supraphysiological) might affect cognitive metabolism and/or performance. Other proposed mechanisms affecting cognition and brain metabolism following endogenous or exogenous ketosis include an increase in blood flow (Svart et al., 2018; Walsh, Caldwell, et al., 2021), stabilization of brain networks (Mujica‐Parodi et al., 2020), modulation of excitatory and inhibitory neurotransmitters (Hone‐Blanchet et al., 2022), and reduced inflammation (see section above). Furthermore, ketone monoester supplementation can preserve or increase plasma brain‐derived neurotrophic factor during hyperglycaemia in adults at normal weight or obesity, respectively (Walsh, Myette‐Cote, et al., 2020), which could conceivably contribute to longer‐term beneficial effects of β‐OHB on metabolic and cognitive functioning.

Emerging evidence implicates a potential beneficial role of exogenous ketosis in various other brain‐related conditions. For example, ingestion of a mix of dextrose and ketone monoester increased the time to fatigue during an exercise test at 55–65% projected wattage at maximum heart rate in individuals with Parkinson's disease compared to ingestion of isocalorically matched dextrose alone (Norwitz et al., 2020). Other preclinical studies showed increased uptake and oxidation of infused β‐OHB following controlled cortical impact (i.e., traumatic brain) injury in adult rats (Prins et al., 2004), and alleviation of alcohol exposure‐induced withdrawal symptoms in mice following consumption of ketone ester alongside regular chow (Bornebusch et al., 2021). Further exploration of the prospects of exogenous ketones for brain health is likely to yield exciting insights from both a basic physiological and an applied therapeutic perspective.

## 6 EXOGENOUS KETONES AND CARDIOVASCULAR FUNCTION

The effect of exogenous ketosis on cardiovascular function – and specifically the heart – is an emerging field of research with most currently available evidence stemming from animal studies. Similar to their function in the brain, ketones can provide an alternative fuel source to the heart under physiological and pathological conditions as demonstrated in animal models using perfused isolated working hearts (Aubert et al., 2016; Ho et al., 2021) and via positron emission tomography following oral ketone ingestion in humans (Cuenoud et al., 2020). In healthy adults, infusion of β‐OHB reduces myocardial glucose uptake compared to infusion of saline without changes in free fatty acid utilization during a hyperinsulinaemic–euglycaemic clamp (Gormsen et al., 2017). However, another study reported myocardial ketone utilization to be uncoupled from the use of other substrates after oral ingestion of a ketone monoester (Monzo et al., 2021). Similar to brain β‐OHB uptake (Blomqvist et al., 1995; Hasselbalch et al., 1995), cardiac β‐OHB extraction was proportionate to β‐OHB delivery and higher in individuals with heart failure compared to healthy controls (Monzo et al., 2021). These data support previous findings of ketones being preferentially utilized for cardiac energy production in advanced heart failure (Bedi et al., 2016) and suggest that the metabolically dysfunctional heart has an increased capacity to metabolize ketones.

As a potential therapy, it has been suggested based on studies in animal models that ketosis can counteract the development of heart failure by reducing inflammation (Byrne et al., 2020). Human clinical studies also support the beneficial effects of exogenous ketones on cardiovascular outcome measures, including reduced blood pressure (Holland et al., 2019; Myette‐Cote et al., 2019), increased cerebral (Svart et al., 2018; Walsh, Caldwell, et al., 2021) and myocardial (Gormsen et al., 2017) blood flow, improved brachial artery flow‐mediated dilatation (Walsh, Neudorf, et al., 2021), and higher cardiac output (Nielsen et al., 2019; Sramko et al., 2022). These studies point to clear cardiovascular effects of exogenous ketones, and future mechanistic and clinical research is needed to evaluate the potential of exogenous ketone supplementation as a therapeutic or preventative option, particularly for individuals living with, or at risk for, heart failure.

Whereas the effects of ketogenic diets on the blood lipid profile (a cardiovascular risk factor) are heavily dependent on the diet's composition (Fuehrlein et al., 2004; Mensink et al., 2003), ingestion of exogenous ketones acutely lowers triglycerides (Liu et al., 2022; Stubbs et al., 2017) with no untoward effects on other blood lipid fractions (Liu et al., 2022; Soto‐Mota et al., 2019). Furthermore, evidence is starting to emerge in preclinical models that supports the ability of β‐OHB to directly affect lipid metabolism (Kemper et al., 2015) and/or the development of atherosclerotic cardiovascular disease via GPR109A (Zhang et al., 2021). However, this area of research remains in its earliest stages and prospective human trials are required to further explore the potential underlying mechanisms.

## 7 CHALLENGES AND FUTURE DIRECTIONS

**FIGURE 2:**
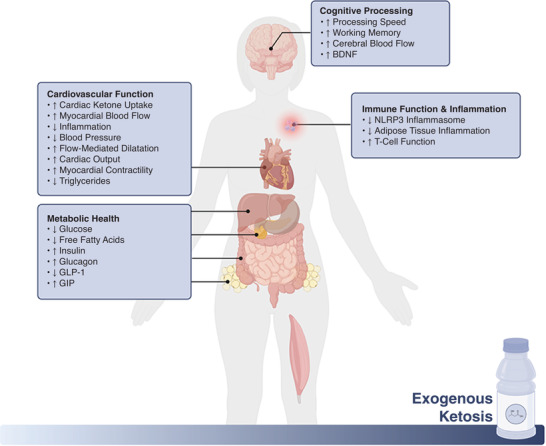
Overview of integrative physiological responses to exogenous ketone supplementation and potential therapeutic effects. BDNF, brain‐derived neurotrophic factor; GIP, glucose‐dependent insulinotropic polypeptide; GLP‐1, glucagon‐like peptide 1. (Created with BioRender.com)

Exogenous ketone supplementation has emerged as a novel tool to help isolate and explore the basic physiology underlying ketosis in various research disciplines and for its therapeutic (Figure 2) potential in clinical settings. Ketone supplements are now commercially available to the general public, being marketed for performance‐enhancing properties such as increased mental clarity, enhanced athletic performance, appetite control and others. However, research on the physiological effects of exogenous ketone ingestion – especially repeated consumption over prolonged periods of time – is still in its infancy and claims such as the above are often unsubstantiated, extrapolated, or even in conflict with existing research and the underlying physiology. Although exogenous ketones can serve as a valuable research tool and hold promise in certain settings, it is important to retain nuance and transparency in scientific discourse and general (e.g., advertising, podcast, etc.) discussions.

One key issue that currently limits clinical or recreational use of exogenous ketones is the general lack of palatability (e.g., bitter taste) and high cost. With more consumer‐directed companies entering the market, ketone supplements have already undergone a transformation from ketone salts that had inherently limited use due to their high mineral content (e.g., leading to gastrointestinal distress, less efficacious at raising blood ketones) to more potent and better‐tolerated ketone monoesters. Novel ketone supplements with different chemical properties (e.g., ketone diesters, different combinations of ketones and precursors) and/or additional ingredients (e.g., flavouring, sweeteners) continue to get developed, but optimizing taste, tolerability and cost remains a challenge. The development of novel ketone supplements also creates the opportunity to refine ketone mixtures with the aim of optimizing plasma kinetics to promote prolonged elevation of blood ketones as opposed to periodic spikes. These new formulations may provide exciting new opportunities for exploring how varying dosing strategies might be fine‐tuned to maximize the benefits of exogenous ketones for different target outcomes.

There are a number of ways to assess the extent of ketosis, the most common of which is the measurement of circulating *R*/d‐β‐OHB concentration in the blood via venous blood draws (usually in laboratory research settings) or capillary finger pricks using handheld monitors. Other ways of non‐invasively assessing ketosis include sporadically measuring acetoacetate in the urine or acetone in the breath. However, novel technologies enabling continuous tracking of ketones similar to continuous glucose monitoring are emerging (Nguyen et al., 2022), and hold potential to help advance our understanding of the physiological effects of exogenous ketone supplementation. Specifically, such technologies could enable characterization of temporal patterns (e.g., circadian, instantaneous) of plasma ketone kinetics that cannot be gleaned from ‘snapshots’ provided by venous blood draws or finger pricks. Additionally, recent findings have started to elucidate the effect of exogenous ketone supplements on the *S*/l‐β‐OHB enantiomer‐specific kinetics (Crabtree et al., 2022; van Rijt et al., 2021), whose role in metabolic function and signalling pathways is incompletely understood. Further research on how exogenous ketones affect the different ketone bodies in different cells or tissues – including in comparison to endogenously induced ketosis – is likely to yield valuable new insights into the physiological properties and metabolic effects of exogenous ketone supplements.

Another promising area of research is the effect of β‐OHB on the brain and central nervous system in general. Teleologically, β‐OHB is often dubbed the ‘starvation molecule’ and much of the brain's vast energy requirements can be met by ketone body oxidation during times of energy deprivation. It therefore stands to reason that β‐OHB might affect aspects of energy homeostasis, appetite regulation, as well as developmental and/or neurological health. Little is currently known about these matters and exogenous ketones will likely serve as a valuable tool to isolate and identify the effects of β‐OHB independent of other metabolic changes occurring during a state of starvation/deprivation‐induced endogenous ketosis.

Finally, further research is warranted to explore the physiological and homeostatic interactions between exogenous ketosis and varying metabolic states, such as during exercise, in the postprandial versus fasted state, in individuals with varying degrees of insulin sensitivity or adiposity, and in conjunction with endogenous ketosis. For example, ketone supplements may have differential effects on immune function or glucose metabolism in young healthy individuals compared to individuals with an acute infection or chronic inflammatory disease. Inhibition of lipolysis induced by exogenous ketone ingestion may be counterproductive when combined with a ketogenic diet, and beneficial effects on cardiovascular outcomes may be balanced by increases in cardiorespiratory stress during exercise (McCarthy et al., 2021).

## 8 CONCLUSION

Exogenous ketone supplements provide a useful tool to perturb metabolic homeostasis for the purpose of studying integrative physiology, while possessing genuine therapeutic potential due to their glucose‐lowering, immunomodulatory, as well as cognitive and cardiovascular effects. This emerging research field promises exciting findings with imminent physiological and therapeutic insight as the knowledge gained from experimental physiological studies is applied to clinical conditions.

## AUTHOR CONTRIBUTIONS

All authors have read and approved the final version of this manuscript and agree to be accountable for all aspects of the work in ensuring that questions related to the accuracy or integrity of any part of the work are appropriately investigated and resolved. All persons designated as authors qualify for authorship, and all those who qualify for authorship are listed.

## CONFLICT OF INTEREST

J.P.L. is volunteer Chief Scientific Officer for the Institute for Personalized Therapeutic Nutrition, a registered charity in Canada. He holds founder shares in Metabolic Insights Inc., a for‐profit company that developed non‐invasive metabolic monitoring devices.
